# Profile and predictor of health-related quality of life among hypertensive patients in south-western Nigeria

**DOI:** 10.1186/1471-2261-9-25

**Published:** 2009-06-17

**Authors:** Michael O Ogunlana, Babatunde Adedokun, Magbagbeola D Dairo, Nse A Odunaiya

**Affiliations:** 1 Department of Epidemiology and Medical Statistics, Faculty of Public Health, College of Medicine, University of Ibadan, Oyo State, Nigeria

## Abstract

**Background:**

The health-related quality of life (HRQOL) of hypertensives may be influenced by blood pressure, adverse effects of drugs used to treat hypertension, or other factors, such as the labelling effect, or beliefs and attitudes about illness and treatment. There is paucity of information on the determinants of HRQOL among black hypertensives especially in the developing countries such as Nigeria. This study describes the HRQOL and its determinants among black patients diagnosed and treated for Hypertension in Nigeria.

**Methods:**

The study was a cross sectional in design that involved 265 hypertensive patients receiving treatment at the medical outpatient unit of the Federal Medical Centre Abeokuta, Nigeria. They were all consecutive patients that presented at the hospital during the period of the study who meet the inclusion criteria and consented to participate in the study. Demographic data, disease characteristics such as symptoms and signs and recent drug history were obtained from the patients and their hospital records as documented by the physician. The SF-36 questionnaire was administered once by interview to the participants to measure their HRQOL. Descriptive statistics was used in summarizing the demographic data and hypertension related histories of the participants. Multiple linear regression was used to model for the influence of socio demographic and clinical variables of the hypertensives on their HRQOL.

**Results:**

Physical functioning domain mean score was far below average (33.53 ± 29.65). Role physical and role emotional domains were a little above average (54.7 ± 40.4, 51.1 ± 40.6 respectively). Role Physical (p = 0.043), Role Emotional (p = 0.003), Vitality (p = 0.014) and Mental Health (p = 0.034) domain mean scores for patients with controlled BP were significantly higher than patients with uncontrolled BP. The overall HRQOL was significantly better in the group of hypertensives with controlled blood pressure (p = 0.014). Increasing blood pressure (p = 0.005) and symptom count (p < 0.001), the presence of stroke (p = 0.008) and visual impairment (p = 0.015) were significant negative predictors of the overall HRQOL.

**Conclusion:**

This study provides evidence for a model that links patients' status with regard to biology (blood pressure), symptoms, and functionality (HRQOL) and may prove useful in guiding follow-up of patients who receive treatment for hypertension. Identification of patient's symptoms, blood pressure, complication/comorbidity and changes in functioning may help clinicians increase their effectiveness in helping patients maintain adherent behaviour with drug and non drug interventions in chronic diseases such as hypertension.

## Background

Hypertension is a condition with tremendous financial and public health impact. The cost of inadequately controlled blood pressure can be measured financially and medically with financial impact in the United States being US$47.2 billion in direct and indirect cost in 2001[1]. The long term medical consequences of untreated hypertension such as myocardial infarction, stroke, congestive heart failure and renal failure are among the most common and serious cause of morbidity and mortality in Nigeria [2].

Although hypertension, especially in mild to moderate stages, is usually considered as an asymptomatic condition, its association with alterations in well-being and health-related quality of life (HRQOL) is still a controversial issue [3]. The relationships between patient, disease, treatment variables, symptoms, and HRQOL were described by Wilson & Cleary [4]. This model proposes that physiologic changes due to illness or treatment, lead to symptoms, which in turn influences functional status or HRQOL. These relationships are influenced by patient and environmental variables that may affect patient perception of symptoms and changes in HRQOL. This general model can be applied to data from clinical studies to ascertain the strength of relationships between HRQOL and patient, disease, and treatment variables. A hypertension diagnosis may increase an individual's awareness of bodily symptoms and make an otherwise "healthy" person ill [5]. The Medical Outcome Study in America found lower general health perception in hypertensive patients compared with those patients without chronic conditions [6]. In a recent population based study, hypertensive individuals were found to have lower health status compared with individuals free from hypertension [7]. Co-morbidity with other diseases associated with hypertension may influence how persons with hypertension rate their HRQOL. In some studies, it has been argued that the low health-related quality of life among those with hypertension is due to subsequent complications of the disease, not to hypertension in itself [5,8].

The profile and predictor of HRQOL of hypertensive patients has been documented in studies from non-black populations ([4,5,9], and [10]). Hypertension is more prevalent in black population and presents with more severe organ complications and accelerated course of hypertension-induced target organ damage [11-14]. These factors may affect the profile and predictor of HRQOL in African hypertensive patients. Other factors that may affect HRQOL of hypertensives are blood pressure, adverse effects of drugs used to treat hypertension, labelling effect, or beliefs and attitudes about illness and treatment ([4,5,9], and [10]). The knowledge of HRQOL of hypertensive patients being a reliable determinant of cerebrovascular diseases (CVDs) events will be useful in reducing the incidence of CVDs [4,5,9,15]. This study documents the HRQOL of hypertensive patients and its predictors in a tertiary health institution located in Abeokuta, south-western Nigeria. Knowledge of any deviation in HRQOL in this population subgroup maybe useful in planning therapeutic interventions that will ensure desirable HRQOL and not just the control of blood pressure.

## Methods

### Study population and design

This study was designed as descriptive cross sectional, documenting HRQOL in patients being managed for hypertension at the medical outpatient clinic of Federal Medical Centre Abeokuta. Participants were all consecutive patients, who had been diagnosed of being hypertensive (recently and formerly diagnosed) receiving treatment at the medical outpatient clinic. The clinic runs four times a week with an average attendance of 200 patients weekly. All hypertensive patients seen at the clinic during the period of this study (three months) who met the inclusion criteria were invited to participate in this study. Sample size was determined using data from a previous study [10], where the SD for HRQOL scores in a similar population of hypertensive patients was 9.3 and assuming an alpha of 0.05 and beta of 0.10 and a 2 tailed test with a difference of a fifth of the SD. It was necessary to involve 263 participants in the study. They were recruited when they presented for Medical Outpatient follow up clinics. Participation in the study was totally voluntary and subjects were asked to complete the questionnaire by interview after their informed consent had been sought and obtained in writing. Ethical approval for the study was given by the Federal Medical Centre Health research committee. The socio demographic factors included in this study were gender, age, ethnic group, marital status, religious affiliation, educational status, social class. Marital status was categorized as married, single and widowed. Educational status was divided into four levels; no education, primary education, secondary education, and tertiary education. Social class was divided into three categories: blue collar workers, white collar workers and self employed [4].

Clinical and disease characteristics included in this study were symptom counts, symptom prevalence, pattern of antihypertensive regimen, co morbidities/complications, body mass index (BMI), self reported duration of hypertension diagnosis, status of hypertension control, height & weight of patient, blood pressure (BP) and mean arterial blood pressure (MABP). Symptom counts [10] were measured as number of symptoms reported by the patients during interview out of the checklist of symptoms commonly observed in hypertension (headaches, fatigue, blurred vision, frequent insomnia, dizziness, facial flushing)[16,17]. Pattern of antihypertensive regimen[18] was divided into six categories: diuretics only, calcium blockers only, angiotensin converting enzyme (ACE) inhibitors only, beta blockers only, centrally acting antihypertensive only and combination therapy. Co morbidities and complications were measured as any other diagnosis reported by the patient other than hypertension. Patient's weight, height and blood pressure were recorded as documented in their hospital records. The average of the three most recent hospital records of these measurements was used for data analysis. Patient's BMI was computed from the weight and height records. MABP [19] was computed from the systolic and diastolic blood pressure records. Status of hypertension control was classified as controlled and uncontrolled hypertension using the 140/90 mmHg criterion [20].

### Measure of health-related quality of life

The Short Form-36 (SF-36) Health Survey, version 2.0 [21] was used to assess HRQOL. Its validity, reproducibility and responsiveness to change over time have been well demonstrated [21]. It has 36 items that measures the health concepts of physical functioning, role limitations due to physical health problems, bodily pain, general health, vitality, social function, role limitations due to emotional problems, and mental health. It also contains a single item that examines change in health over time. Summary measures of physical health (Physical Component Summary [PCS]), mental health (Mental Component Summary [MCS]) and Total QOL score (T-score) were derived from the completed questionnaire. This instrument was translated to Yoruba language (the local language of the study location) by an expert linguist. Forward and backward translation was done during this translation to ensure content validity. The Yoruba Language version was administered to respondents who were not proficient in English language. The SF-36 was administered once by interview to the participants. Estimated duration for administration of the instrument was 15 minutes. Three trained research assistants were employed to administer the questionnaires and obtain relevant clinical information from the respondents by clinical history and from the hospital case files of the respondents.

### Statistical analyses

Descriptive statistics was used in summarizing the demographic data and Hypertension related histories of the participants. The association between QOL scores, socio demographic and clinical variables were determined using stepwise multiple linear regression analyses with QOL score as the outcome variable, while socio demographic and clinical variables were predictor variables. Duration of hypertension diagnosis, age and MABP of respondents were used as continuous predictor variables while other non-linear categorical predictor variables were dichotomized for the purpose of the multiple linear regressions. The *p*-value for addition to the model was *p *< 0.05 and for removal from model *p *> 0.1. A *p *< 0.05 was considered statistically significant; confidence interval was calculated for the β-estimate.

## Results

Three hundred hypertensive patients were invited to participate in this study out of which 265 participated thus giving a response rate of 85.3%. Twenty five (8.3%) patients declined to participate due to personal reasons while 10 (3.3%) patients did so due to lack of interest. The mean age of the 265 hypertensive patients who participated was 62.4 ± 10.7 years. Less than half of the respondents (43.4%) were male and 32.1% had tertiary education. About three-quarter (76.2%) of the respondents were married and 97.7% of them were of the Yoruba tribe. Few of them (8.3%) were blue collar workers and 66.4% of the respondents were Christians. Although 50.6% had no symptoms, the most prevalent symptom reported was frequent insomnia in 37% of respondents. Diabetes Mellitus was the most frequent co morbidities/complication in 23.6% of respondents. Anti-hypertensive medication prescribed was mainly combination therapy in 65.7% of respondents. The mean duration of hypertension diagnosis for respondents was 4.7 ± 6.1 years. Blood pressure was controlled in 52.1% of the respondents (that is systolic and diastolic blood pressure was less than 140/90 mmHg). Table 1 and 2 shows the respondents socio-demographic and clinical characteristics respectively.

**Table 1:** Socio-demographic characteristics of respondents

Classification	Characteristics	Frequency (Total = 265)	Percentage
Gender	Male	115	43.4
	Female	150	56.6
Marital Status	Single	8	3
	Married	202	76.2
	Widowed	55	20.8
Ethnic Grouping	Yoruba	257	97
	Ibo	8	3
Religious Affiliation	Christianity	176	66.4
	Islam	88	33.2
	Traditional	1	0.4
Educational Status	None	76	28.7
	Primary	42	15.8
	Secondary	62	23.4
	Tertiary	85	32.1
Social Class	White Collar	99	37.4
	Self Employed	91	34.3
	Blue Collar	22	8.3
	Missing	53	20
Age	30–39	3	1.1
	40–49	22	8.3
	50–59	70	26.4
	>60	162	61.1
	Unknown age	8	3.0

**Table 2:** Clinical variables of the respondents

**clinical variables**	Frequency	Percentage
**Symptom counts**		
No symptom	134	50.6
1 symptom	88	33.2
2 symptoms	23	8.7
>3 symptoms	20	7.5
**Comorbidities/complications**		
Angina Pectoris	5	1.9
Diabetes	63	23.6
Nephropathy	2	0.8
Stroke	10	3.9
Myocardial Infarct	1	0.4
Heart failure	6	2.3
Visual impairment	34	12.8
**Body mass index BMI (kg/m^2^)**		
<19	19	7.2
19 – 25	140	52.7
26 – 30	54	20.5
31 and above	52	19.7
**Duration of hypertension (years.)**		
0.3–3.9	173	65.3
4.0 – 10.9	55	20.9
11 – 30	37	13.8
**Prevalence of symptoms**		
Headache	28	10.6
Fatigue	21	7.9
Blurred vision	25	9.4
Frequent Insomnia	98	37.0
Dizziness	16	6.0
Facial Flushing	12	4.5
Tinnitus	16	6.0

Table 3 shows the mean and standard deviation of respondents' quality of life (QOL) score by domains. The Physical Functioning domain mean score was far below average. Role Physical and Role Emotional domains were a little above average. Other domains were far above average. Role Physical (p = 0.043), Role Emotional (p = 0.003), Vitality (p = 0.014) and Mental Health (p = 0.034) domain mean scores for patients with controlled BP were significantly higher than patients with uncontrolled BP. Table 4 shows the comparison of mean QOL summary measures score for respondents with controlled and uncontrolled blood pressure. There was a significant difference between the Mental Component summary (MCS) score (p = 0.004) and Total (T) QOL (p = 0.014) score between the two groups of hypertensive patient with respondents who have controlled blood pressures having higher mean scores. Tables 5, 6 and 7 show the variables that had significant influence on the QOL summary scores.

**Table 3:** Mean and standard deviation of Respondents' (QOL) domain scores

	Mean ± SD			P-value
		
Domains	Total	Patients with Controlled BP	Patients with Uncontrolled BP	
Physical Functioning	33.53 ± 29.65	32.25 ± 29.73	34.93 ± 29.63	0.464
Role Physical	54.66 ± 40.36	59.49 ± 41.47	49.41 ± 38.59	0.043*
Role Emotional	51.14 ± 40.58	58.21 ± 39.48	43.39 ± 40.51	0.003*
Vitality	79.85 ± 14.57	81.94 ± 14.19	77.55 ± 14.18	0.014*
Mental Health	70.65 ± 17.87	72.86 ± 17.48	68.20 ± 18.03	0.034*
Social Functioning	75.19 ± 22.97	75.63 ± 24.09	74.70 ± 21.76	0.743
Bodily Pain	76.28 ± 30.17	77.54 ± 28.86	74.90 ± 31.60	0.479

* = significant at < 0.05

**Table 4:** Comparison of Mean QOL score for Respondents with Controlled and Uncontrolled Blood Pressure

	Mean ± SD			
	
	Total	Controlled BP	Uncontrolled BP	p-value
Physical component summary (PCS) score	56.54 ± 15.75	57.83 ± 15.53	55.13 ± 15.93	0.164
Mental component (MCS) score	69.21 ± 17.66	72.16 ± 18.18	65.01 ± 16.53	0.004*
Total quality of life (T-QOL) score	62.74 ± 15.61	65.01 ± 15.66	60.28 ± 15.24	0.014*

* = significant at < 0.05

**Table 5:** Influence of variables on Physical component summary (PCS) score

Variable	Sub-group	Mean ± SD	Test statistic value	p-value
Symptom count	No symptom	51.62 ± 13.99	10.71^b^	< 0.001*
	≥ 1 symptom	61.63 ± 15.96		
BMI	Obese	51.36 ± 16.96	-2.66^b^	0.008*
	Not Obese	57.79 ± 15.25		
Visual impairment	Yes	51.84 ± 12.76	-2.07^b^	0.04*
	No	56.95 ± 16.19		

b = t-value, * = significant at < 0.05

**Table 6:** Influence of variables on Mental Component Summary (MCS) score

Variable	Sub-group	Mean ± SD	Test statistic value	p-value
Education status	Any Educ.	71.80 ± 17.09	-3.89^b^	< 0.001*
	No Educ.	62.66 ± 17.47		
Marital status	Married	70.75 ± 17.26	2.59^b^	0.01*
	Single	64.17 ± 18.14		
Occupational class	White collar	72.42 ± 16.67	4.47^a^	0.01*
	Self empl.	65.94 ± 18.37		
	Blue collar	63.00 ± 18.46		
BMI	Obese	62.82 ± 16.81	2.90^b^	0.004*
	Not obese	70.71 ± 17.59		
Symptom count	No symptom	64.78 ± 15.39	-4.29^b^	< 0.001*
	≥ 1 symptom	73.86 ± 18.73		
Stroke	Yes	52.68 ± 16.42	-2.99^b^	0.003*
	No	69.49 ± 17.43		
Visual impairment	Yes	56.93 ± 14.99	-4.80^b^	< 0.001*
	No	70.63 ± 17.34		

a = f-value, b = t-value, * = significant at < 0.05

**Table 7:** Influence of variables on T – QOL score

Variable	Sub-group	Mean ± SD	Test statistic value	p-value
Education status	Any Edu.	64.41 ± 14.85	-2.78^b^	0.006*
	No Edu.	58.59 ± 16.76		
BMI	Obese	56.49 ± 16.80	-3.27^b^	0.001*
	Not Obese	64.25 ± 14.99		
Symptom count	No symptom	58.20 ± 12.98	-5.03^b^	< 0.001*
	≥ 1 symptom	67.46 ± 16.76		
Stroke	Yes	51.31 ± 11.67	-3.03^b^	0.01*
	No	62.88 ± 15.68		
Visual impairment	Yes	54.38 ± 12.11	-3.23^b^	< 0.001*
	No	63.63 ± 15.79		

b = t-value, * = significant at < 0.05

Table 8 shows the result of multivariate analysis relating MCS score to predictor variables. Increasing MABP (p = 0.013), increasing symptom count (p < 0.001), the presence of Stroke (p < 0.001) and visual impairment (p = 0.005) were significant negative predictors of the MCS score. Educational status (p = 0.040) is a significant positive predictor of MCS score. Table 9 shows the result of multivariate analysis relating PCS score to predictor variables. Increasing BMI (p = 0.005) and symptom count (p < 0.001) are significant negative predictors of the PCS score. Multivariate analysis relating T-QOL score with predictor variables revealed that increasing MABP (p = 0.005) and symptom count (p < 0.001), and the presence of Stroke (p = 0.008) and visual impairment (p = 0.015) were significant negative predictors of HRQOL (Table 10).

**Table 8:** Multiple regression relating MCS score to predictor variables

MCS	Coefficient	Beta	p-value	Lower CL	Upper CL
Constant	16.854		0.459	-27.952	61.660
Age	-0.124	-0.075	0.291	-0.356	0.108
Edu. status	6.261	0.158	0.040*	0.281	12.241
Marital status	-0.166	-0.004	0.953	-5.772	5.439
Occupational class	0.379	0.014	0.852	-3.632	4.391
BMI	-0.298	-0.095	0.188	-0.742	0.147
MABP	-0.286	-0.170	0.013*	-0.511	-0.060
HTN duration	0.109	0.039	0.578	-0.277	0.496
Symptom count	12.035	0.339	0.000*	7.190	16.881
Stroke	21.552	0.250	0.000*	10.166	32.939
Visual impairment	12.599	0.195	0.005*	3.832	21.365

* = significant at < 0.05

**Table 9:** Multiple regression relating PCS score to predictor variables

PCS score	Coefficient	Beta	p-value	Lower CL	Upper CL
Constant	44.551		0.000	24.963	64.139
BMI	-0.517	-0.180	0.005*	-0.879	-0.155
Symptom count	10.546	0.335	0.000*	6.542	14.550
Visual impairment	5.380	0.890	0.155	-2.050	12.810
HTN Duration	-0.152	-0.059	0.359	-0.477	0.174

* = significant at < 0.05

**Table 10:** Multiple regression relating T-QOL score to predictor variables

T-QOL	Coefficient	Beta	p-value	Lower CL	Upper CL
Constant	28.423		0.100	-5.457	62.304
Edu. status	3.030	0.090	0.230	-1.940	7.999
Marital status	0.327	0.009	0.892	-5.071	4.417
Occupational class	-0.263	-0.011	0.880	-3.702	3.177
BMI	-0.252	-0.093	0.179	-0.621	0.117
MABP	-0.273	-0.188	0.006*	-0.466	-0.080
HTN duration	-0.040	-0.017	0.814	-0.295	0.375
Symptom count	12.284	0.404	0.000*	8.169	16.399
Stroke	13.551	0.179	0.008*	3.600	23.501
Visual impairment	9.411	0.167	0.015*	1.823	16.399

* = significant at < 0.05

## Discussion

Many studies [4,5,9,10] have analysed HRQOL among persons with hypertension using a comprehensive generic instrument and with a multivariate approach to identify association that are independent of socio-demographic factors. However these studies are from developed countries and may not give a true picture of the profile of HRQOL in hypertensives in a developing country like Nigeria. In this study, the impact of hypertension on the dimensions in the SF-36 while adjusting for socio demographic and clinical characteristics of the hypertensive patient was investigated. The obtained data provide information on HRQOL across a range of domains among individuals receiving treatment for hypertension in a tertiary health centre.

### Profile of HRQOL

In this study the Physical Functioning domain mean score was far below average (33.53 ± 29.65). Role Physical (presence of physical problems) and Role Emotional (presence of emotional problems) domains were a little above average (54.66 ± 40.36, 51.14 ± 40.58 respectively). Other domains were far above average. These domain scores are far lower than those presented by Bardage & Isacson [4], (Physical functioning, 70.90 ± 27.40; Role physical, 66.50 ± 41.50; Role emotional, 77.20 ± 37.40). This shows that on the average the HRQOL of the population in this study is lower than that from the study by Bardage & Isacson [4] which was a population based study of hypertensives but this study is a hospital based study. The differences in HRQOL scores can be due to the fact that hypertensives seen at hospitals have more health limitation compared to those seen in the general population. Furthermore, the study population are black hypertensive patients; and hypertension has been said to be more prevalent with severe complications in this population [11-14] hence a worse profile of HRQOL than non black population. Also extrinsic factors like the differences in development and economic strength of the two study populations may explain the differences in the HRQOL but this study did not explore this fact.

### HRQOL and Control of blood pressure

The QOL of hypertensives who had controlled blood pressure and those with uncontrolled blood pressure was compared. The physical component summary (PCS) score of the QOL was not significantly different (p = 0.16) between the two groups of hypertensives but the mental component summary (MCS) score and the total T-QOL score were significantly different (p = 0.004 & 0.014 respectively) for the two groups of hypertensives. Shapiro et al[22], reported behavioural impairments in patients with elevated blood pressure and Mena-Martins et al [5], and Wie et al [9], showed that the HRQOL of hypertensives improve when control of blood pressure is achieved. This study also shows that the MCS and T-QOL score of hypertensive who had controlled blood pressure was significantly higher than those with uncontrolled blood pressure hence better quality of life. Elevated blood pressure is mostly without physical symptoms [17] as seen in more than half of the respondents; this may explain why PCS score of hypertensives with controlled and uncontrolled blood pressure was not significantly different.

### Predictors of HRQOL

Results from multiple regression analysis revealed that a hypertensive that has a low blood pressure (MABP less than 108), no symptom, any form of education, and does not have a stroke or visual impairment would have a better mental component of HRQOL than any hypertensive who does not have these characteristics. A hypertensive patient who has BMI less than 30 (not obese) and no symptom will have a better physical component of HRQOL than any hypertensive patient who does not have these characteristics. Also, a hypertensive patient who has blood pressure (MABP) less than108, no symptom and who does not a have stroke or visual impairment will have a better overall HRQOL than a hypertensive who does not have these characteristics. The adverse influence of increasing blood pressure on HRQOL has been well documented in literature[4,5,9,15,23] but very few studies[10] have emphasized the effects of symptoms on HRQOL of hypertensive patients. Though hypertension is seen as an asymptomatic condition, increasing symptom count and blood pressure is a major determinant of the HRQOL of hypertensives. Erickson et al[10] concluded that symptom count and symptom distress score are the major predictors of HRQOL in hypertensives. Evidence from this study shows that symptom count and blood pressure are the major predictors of HRQOL of hypertensive patients. This result does not essentially contradict the study by Erickson et al[10], since increasing blood pressure produces increasing symptoms and distress in hypertensive patients. Symptom monitoring and management are central to the improvement of patients' HRQOL. The adverse effects of having a stroke on HRQOL of hypertensive patients has been well documented[4,5,9] but the effects of visual defect as a comorbidity/complication on the HRQOL of hypertensive patients has not been commonly reported. The presence of a stroke and a visual impairment increases the extent of disability in hypertensive patients hence worsening their HRQOL.

The data from this study may be subject to Berksonia's bias as the respondents were all hypertensives who presented in the hospital with specific complaint. In other to make generalizable conclusions, a population based study using a probability sampling technique will be recommended to survey the HRQOL of hypertensives at other meeting points apart from the hospital. This may give a more objective description of their HRQOL. Cross cultural content validity of the SF-36 questionnaire was assumed in this study. This presents a major limitation when comparing the concept of quality of life in different cultures as the respondents understanding of the questions might vary. This bias was minimized by ensuring that the interviewers were trained in the questionnaire administration and the questionnaire was translated into the local language of the study location with forward and backward translation.

Nevertheless this study provides evidence for a model that links patients' status with regard to biology, symptoms, and functionality (HRQOL) and by implication may prove useful in guiding follow-up of patients who receive treatment for hypertension. Identification of patient's symptoms, complication/co morbidity and changes in HRQOL will help clinicians increase their effectiveness in helping patients maintain adherent behaviour with drug and non drug interventions in chronic diseases such as hypertension. Findings from this study shows that the predictors of HRQOL (blood pressure, symptoms and co morbidities) in black hypertensive patients are not essentially different from those reported in non-black population although the co morbidities and complications in question are diverse.

## Conclusion

Results from this study revealed that none of the socio-demographic and clinical characteristics of the respondents significantly affected their control of blood pressure, but the HRQOL was significantly better in the group of hypertensives that had controlled blood pressure. Blood pressure and symptoms counts are major predictors of HRQOL in hypertensives. The presence of Stroke and Visual defect as a co morbidity or complication are also major determinant of the HRQOL of hypertensives.

## Competing interests

The authors declare that they have no competing interests.

## Authors' contributions

MOO participated in the acquisition of the data and conceptualisation of the study, performed statistical analysis, and drafted the manuscript; BA & MDD reviewed data analysis results and critically revised the manuscript. NAO participated in the conceptualisation of the study and critically revised the manuscript. All authors read and approved the final manuscript.

## Pre-publication history

The pre-publication history for this paper can be accessed here:


